# A hint for the obesity paradox and the link between obesity, perirenal adipose tissue and Renal Cell Carcinoma progression

**DOI:** 10.1038/s41598-022-24418-9

**Published:** 2022-11-19

**Authors:** José Preza-Fernandes, Pedro Passos, Miguel Mendes-Ferreira, Adriana R. Rodrigues, Alexandra Gouveia, Avelino Fraga, Rui Medeiros, Ricardo Ribeiro

**Affiliations:** 1 Urology Department, Hospital Senhora da Oliveira, Creixomil, Portugal; 2 Tumour and Microenvironment Interactions Group, i3S, Instituto de Investigação e Inovação Em Saúde da Universidade Do Porto, Rua Alfredo Allen, 208, 4200-135 Porto, Portugal; 3 ICBAS, Abel Salazar Biomedical Sciences Institute, University of Porto, Porto, Portugal; 4 Ageing & Stress Group, i3S - Instituto de Investigação e Inovação Em Saúde, University of Porto, Porto, Portugal; 5 Departamento de Biomedicina, Unidade de Biologia Experimental, Faculdade de Medicina da Universidade Do Porto, Porto, Portugal; 6 Faculdade de Ciências da Nutrição e Alimentação, Universidade Do Porto, Porto, Portugal; 7 Department of Urology, Centro Hospitalar e Universitário Do Porto, Porto, Portugal; 8 Molecular Oncology Group, Portuguese Institute of Oncology, Porto, Portugal; 9 Laboratory of Genetics and Instituto de Saúde Ambiental, Faculdade de Medicina, Universidade de Lisboa, Lisboa, Portugal; 10 Department of Pathology, Centro Hospitalar Universitário Do Porto, Porto, Portugal

**Keywords:** Cancer, Cell biology, Biomarkers, Urology

## Abstract

Increasing evidence supports a role for local fat depots in cancer outcomes. Despite the robust positive association of obesity with renal cell carcinoma (RCCa) diagnosis, increased adiposity is inversely related to RCCa oncological outcomes. Here, we sought to ascertain whether imagiologically assessed local fat depots associate with RCCa progression and survival and account for this apparent paradox. A retrospective cohort of renal carcinoma patients elective for nephrectomy (n = 137) were included. Beyond baseline clinicopathological characteristics, computed tomography (CT)-scans at the level of renal hilum evaluated areas and densities of different adipose tissue depots (perirenal, subcutaneous, visceral) and skeletal muscle (*erector spinae, psoas* and *quadratus lumborum* muscles) were analyzed. Univariate and multivariable Cox proportional hazards models were estimated following empirical analysis using stepwise Cox regression. Age, visceral adipose tissue (VAT) area and body mass index (BMI) predicted tumour-sided perirenal fat area (*R*^*2*^ = 0.584), which presented upregulated *UCP1* expression by 27-fold (*P* = 0.026) and smaller adipocyte areas, compared with subcutaneous depot. Multivariate analyses revealed that increased area of perirenal adipose tissue (PRAT) on the contralateral and tumour side associate with improved progression-free survival (HR = 0.3, 95CI = 0.1–0.8, *P* = 0.019) and overall survival (HR = 0.3, 95CI = 0.1–0.7, *P* = 0.009). PRAT measurements using CT, might become a possible tool, well correlated with other measures of obesity such as VAT and BMI, that will improve determination of obesity and contribute to assess the risk for disease progression and mortality in renal cancer patients. Present data supports the obesity paradox in RCCa, assumed that larger PRAT areas seem to protect from disease progression and death.

## Introduction

Obesity is a worldwide health problem, commonly associated with increased risk and worse prognosis for a wide range of malignancies^1,2^. It is a well-established risk factor for developing renal cell carcinoma (RCC), particularly the clear cell histological subtype^3^, although a protective role in prognosis has been described^4^. The biological links underlying the association between obesity and kidney cancer, particularly its putative protective role in progressing disease or death, remain poorly understood. Furthermore, concern exists regarding the method used to assess excess adiposity^5^. While BMI is the mostly used measure, it is an imperfect estimate of adiposity, particularly in men because of their greater lean mass^6^. Increasingly used tools include visceral and subcutaneous adipose tissue area and radiodensity measurements, using cross-sectional abdominal images from computed tomography or magnetic resonance (which are appropriate in clinical context and provide useful local depots adiposity information). These, together with perirenal adipose tissue (PRAT) thickness have been well-correlated with worst outcomes in oncological settings^7–10^. Besides adiposity, skeletal muscle status on CT or MRI imaging may provide useful information for prognosis, at least in metastatic renal cancer patients, once high muscle density was independently associated with improved outcomes^11^. Indeed, muscle wasting has been linked to worse prognosis in patients with various types of urological cancers^12^.

In recent years, data from several cancer models supports a local, depot-specific crosstalk of adipose tissue with malignant cells in tumour microenvironment, mediated by cells and/or soluble factors^13^. Notably, the transcriptome and proteome of distinct adipose tissue depots differs among anatomical origins^14–16^. Previous reports aimed to characterize PRAT suggest it is mainly composed of brown-like or beige adipose tissue^17^. While the role of the PRAT depot in the crosstalk with tumour microenvironment in non-metastatic kidney cancer continues to be disclosed, recent contributions from tumour and perirenal fat transcriptomics and PRAT in vitro studies^18–20^ strengthened our understanding of renal cancer-associated obesity paradox.

Here, we sought to uncover whether perirenal adiposity (area and radiodensity of perirenal adipose tissue depot and of skeletal muscle) associates with other measures of obesity and is related with renal cell carcinoma (progression free survival and overall survival).

## Patients and methods

### Patients

This retrospective cohort study included patient’s elective for partial or radical nephrectomy and with histologically confirmed renal cell carcinoma (RCCa). From a total of 229 subjects, 137 were enrolled, (92 were excluded from further analysis due to missing data, lost to follow-up, concomitant malignancies, and infrequent histopathological subtypes). Subjects were recruited at diagnosis or during follow up in the Departments of Urology of Santo António Hospital in Porto and Senhora da Oliveira Hospital in Guimarães between January 2007 and December 2016. Complete medical record data with follow up information was obtained from clinical charts. Aggressiveness and prognosis variables included TNM pathological staging, Fuhrman grade, and the risk of disease progression was classified according to SSIGN score for ccRCC cases.

To further characterize and assemble pilot data on gene expression from PRAT and subcutaneous adipose tissue (SCAT), samples were collected from 9 renal cancer patients submitted to elective radical nephrectomy at Centro Hospitalar do Alto Ave, Portugal. SCAT (approximately 2–3 g) was removed at the place of laparoscopic port or subcostal incision, and PRAT was collected inside the Gerota fascia, at the level of each tumour (respecting oncological surgery safety limits). Immediately upon surgical removal, adipose tissue samples were included in RNA*later* tissue stabilization solution (Thermo Fisher Scientific Inc, Carlsbad, CA, USA).

All methods were carried out in accordance with relevant guidelines and regulations. The retrospective study was conducted on already available data, for which formal consent was difficult to obtain. Ethical approval was waived by the local Ethics Committee of participating Hospitals [Centro Universitario do Porto—Porto 2021–127(103-DEFI/107-CE) and Hospital Senhora da Oliveira—Guimarães (Ref 17/2015)] in view of the retrospective nature of the study and all the procedures being performed were part of the routine care. Informed consent was obtained from individual participants from which biological samples were collected.

### Anthropometric and imagiological methods

Anthropometry was assessed using the BMI (weight in kilograms/height in meters^-2^), classified in agreement with the WHO recommendation^21^.

PRAT, visceral adipose tissue (VAT) and skeletal muscle areas and radiodensity measurements were performed on routine computed tomography (CT) scans undertaken on participants at the time of diagnosis and before surgery. Measurements were made by a urological surgeon (JPF). Cross-sectional areas plus radiation attenuation (in Hounsfield units, HU) of PRAT and VAT and skeletal lumbar muscle were measured at the level of the renal hilum in the tumour and contralateral sides (from CT images using the Sectra workstation IDS7 ® software). Briefly, the PRAT areas were delimited externally at the level of Gerota fascia, internally by the kidney parenchyma and medially by the renal vasculature (if a tumour was occupying the perirenal space on CT slice, its area was excluded), whereas the VAT included the selected area inside the abdominal wall (behind abdominal transversus muscle) without viscera. Skeletal muscle area included quantification of the erector spinae, psoas and quadratus lumborum muscles (examples of the measurement of areas are depicted in Fig. 5).

### Gene expression

Total RNA was extracted from adipose tissue using the Triplextractor direct RNA kit (Grisp, Porto, Portugal), Briefly, 200 mg of adipose tissue was homogenized in Triplextractor solution (Grisp) with magnetic BulkBeads (Precellys, Montigny-le-Bretonneux, France) using the MagNA Lyser Instrument (Roche, Mannheim, Germany). RNA was purified with a spin column system following the manufacturer's instructions (Grisp). First-strand cDNA was synthesized with 1 ug of total RNA, using the NZY First-Strand cDNA Synthesis kit (Nzytech, Lisboa, Portugal). The transcript levels of *UCP1, DIO2 and PRDM16* were quantified in duplicate by quantitative real time polymerase chain reaction (qPCR) on a StepOne Real-Time PCR System (Applied Biosystems, Foster City, CA, USA) using SYBR® Select Master Mix (Applied Biosystems) and specific primers (Supplementary table 4). The gene *TBP* was used as internal control. Relative quantification between PRAT versus SCAT was calculated using the REST 2009 software (Qiagen, Hilden, Germany).

### Adipocyte size evaluation

PRAT and SCAT from 7 subjects were formalin-fixed overnight, embedded in paraffin and sectioned at a thickness of 5 μm. Paraffin-embedded tissue sections were deparaffinized, rehydrated and stained with Mayer’s Hematoxylin following by 0.1% eosin solution. Afterwards, tissue sections were dehydrated and mounted in Entellan media (Merk, Darmstadt, Germany). Stained adipose tissue was imaged in a light microscope Olympus DP25 Camera Software Cell B (200x), and more than 10 random microphotographs captured for each patient. Image J was used for adipocyte reconstruction and measurement of adipocyte area in 100–200 cells per subject, from multiple slides. Pixel areas of all individual cells were averaged by size interval for each patient.

### Statistics

Descriptive analyses included absolute count and frequencies, median with respective inter-quartile range (IQR) and mean ± standard error of mean (SEM). Departure from normality was analyzed using Shapiro–Wilk test. The Wilcoxon test was used for comparisons between areas and radiodensities of tumour and contralateral PRAT depots. Adipocyte area’s differences among adipose tissue depots were calculated using Mann–Whitney test.

Continuous independent variables such as CT measures (VAT area, PRAT areas and radiodensities, and skeletal muscle area and radiodensity) were stratified into tertiles. Then, a Pearson chi-square test was used to test the association of age, gender, tobacco, and hypertension with tertiles of CT measures (areas, ratios and radiodensities of adipose tissue and skeletal muscle). Moreover, a logistic regression with adjustment for age and gender was done to assess the association between obesity measures and RCCa aggressiveness variables: stage and grade of disease. The primary and secondary endpoints were progression-free survival (PFS) and overall survival (OS), respectively. Survival analyses included initial empirical time-to-event and comparison of Kaplan–Meier estimates for both primary and secondary endpoints (progression-free survival and overall survival), to test the robustness of independent variables, both clinicopathological and CT measures, for explaining the outcomes. Then, the variables with *P* < 0.10 on univariate analysis were included in multivariate stepwise Cox regression (P for retention 0.05) to further refine the strength of association of the toughest variables. Finally, a Cox proportional-hazards model was fitted, and a proportional-hazards assumption test (based on Schoenfeld residuals) run, including only the lasting variables. The best Cox regression models for PFS and OS was then fitted to evaluate whether the predictive and prognostic value was maintained in the subgroup analysis including only Clear Cell RCCa (n = 91). Statistical analyses were conducted in STATA 12.0. Graphics were created in GraphPad Prism 8.0.1. Significance was attributed when P-value < 0.05.

## Results

### Descriptive demographic, clinicopathological and imagiological measures data in the RCCa cohort

Descriptive clinicopathological characteristics of the participants are summarized in Table 1. The descriptive statistics of CT imaging measures at diagnosis, included areas of VAT, bilateral PRAT, and skeletal muscle, ratios of PRAT adjusted by kidney area, and radiodensity of PRAT depots and skeletal muscle from our cohort of renal cell cancer (RCCa) patients is depicted in Supplementary table 1.

**Table 1:** Baseline clinicopathological characteristics of renal cell cancer patients (n = 137) included in the study.

Variables	n (frequency)
Age at diagnosis, yrs*	
< 64.7	68 (49.6)
> 64.7	69 (50.4)
**Gender**	
Male	87 (63.5)
Female	50 (36.5)
**BMI categories (WHO)**	
Normal weight, < 25 kg.m^2^	50 (36.5)
Overweight, 25–30 kg.m^2^	52 (38.0)
Obesity, > 30 kg.m^2^	35 (25.5)
**Tobacco**	
No	84 (61.3)
Yes	53 (38.7)
**Hypertension**	
No	51 (37.2)
Yes	86 (62.8)
**Stage of disease**	
I—II	109 (0.80}
III—IV	28 (0.20)
**Surgery**	
Radical nephrectomy	80 (58.4)
Partial nephrectomy	57 (41.6)
**Surgical margins**	
Negative	130 (94.9)
Positive	7 (5.1)
**Histology**	
Clear cells	91 (66.4)
Papillary I and II	20 (14.6)
Chromophobe	16 (11.7)
Other	10 (7.3)
**Furhman grade**	
Low grade (1–2)	109 (79.6)
High grade (3–4)	28 (20.4)
**Tumor size, cm**	
< 5	83 (60.6)
> 5	54 (39.4)

*The cut point was median age; WHO, World Health Organization.

### Association among clinicopathological variables and tertiles of adiposity and skeletal muscle measures

We evaluated whether demographic and clinical variables known to influence the natural history of RCC, such as age, gender, tobacco, and hypertension, were associated with tertiles of fat and skeletal muscle measures (Supplementary table 2). The top tertiles of VAT and PRAT areas, as well as kidney-adjusted PRAT areas, were associated with male gender and with arterial hypertension (*P* < 0.005). Moreover, there was an overrepresentation in top tertiles of skeletal muscle (SkM) radiodensity in younger and normotensive (*P* < 0.0001 and *P* = 0.014, respectively), and of PRAT radiodensity in tobacco consumers and normotensive participants (*P* = 0.002 and *P* = 0.047, respectively).

### Assessment of the relationship between PRAT areas with other adiposity measures

**Figure 1:**
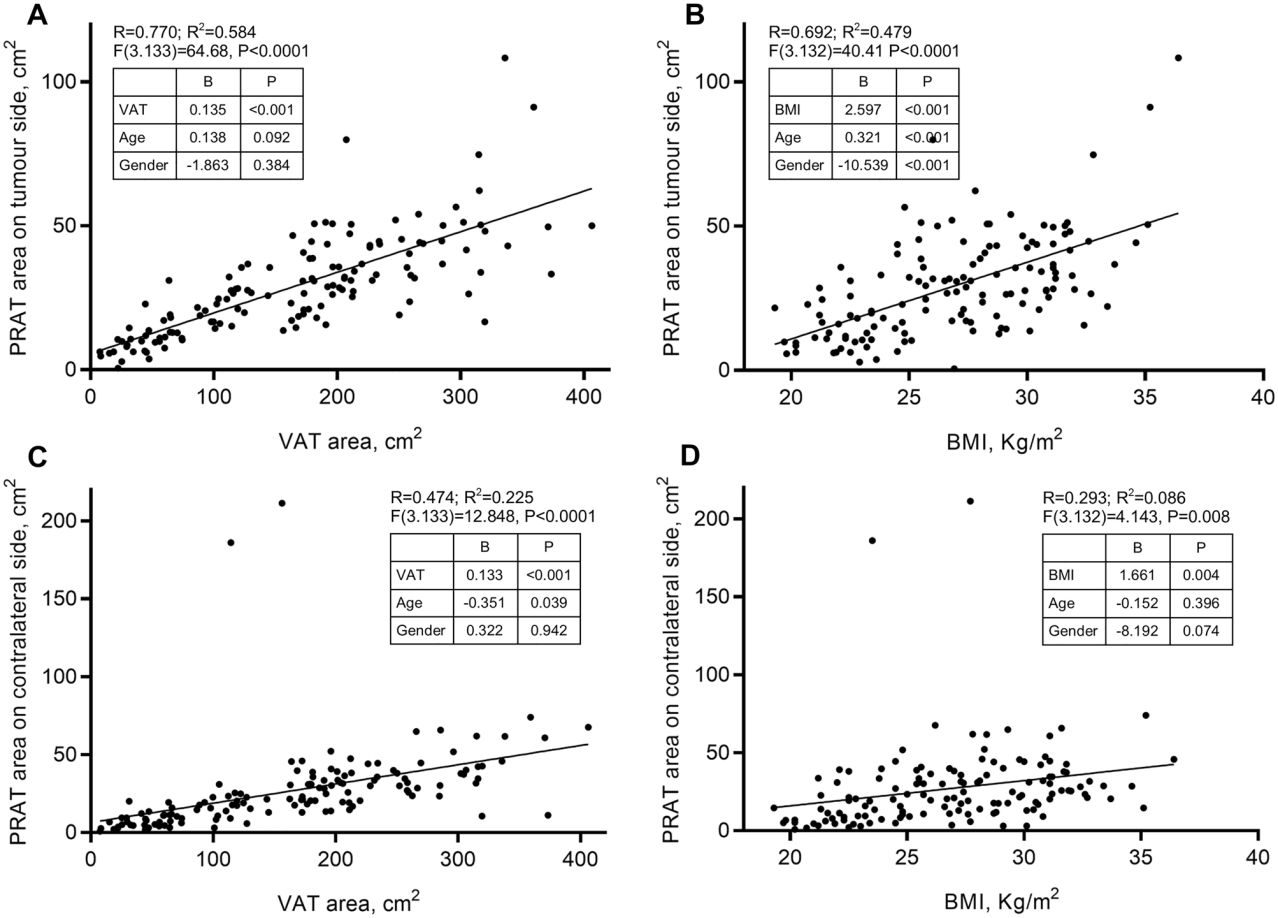
Association of overall adiposity measures with perirenal adipose tissue areas. Linear regression with adjustment for age and gender, was used to calculate R and R^2^ between either VAT or BMI and tumour-sided and contralateral PRAT. Association between PRATt area with VAT (**A**) and BMI (**B**), and between PRATcl area with VAT (**C**) and BMI (**D**). VAT, visceral adipose tissue; PRAT, perirenal adipose tissue; BMI, body mass index. B, B-coefficient from linear regression analysis; P, *P*-value.

The relationship between obesity variables (VAT area and BMI), adjusted by age and gender, with tumour side and contralateral PRAT area is depicted in Fig. 1. Two overall multivariable linear regression were run to predict tumour and contralateral PRAT area from gender, age, BMI and VAT area. The independent variables predicted tumour-sided PRAT area better than contralateral PRAT area (*R*^*2*^ = 0.619 and *R*^*2*^ = 0.224, respectively).

### Comparison of PRAT areas and radiodensities between tumour-bearing and tumour-free kidneys

**Figure 2:**
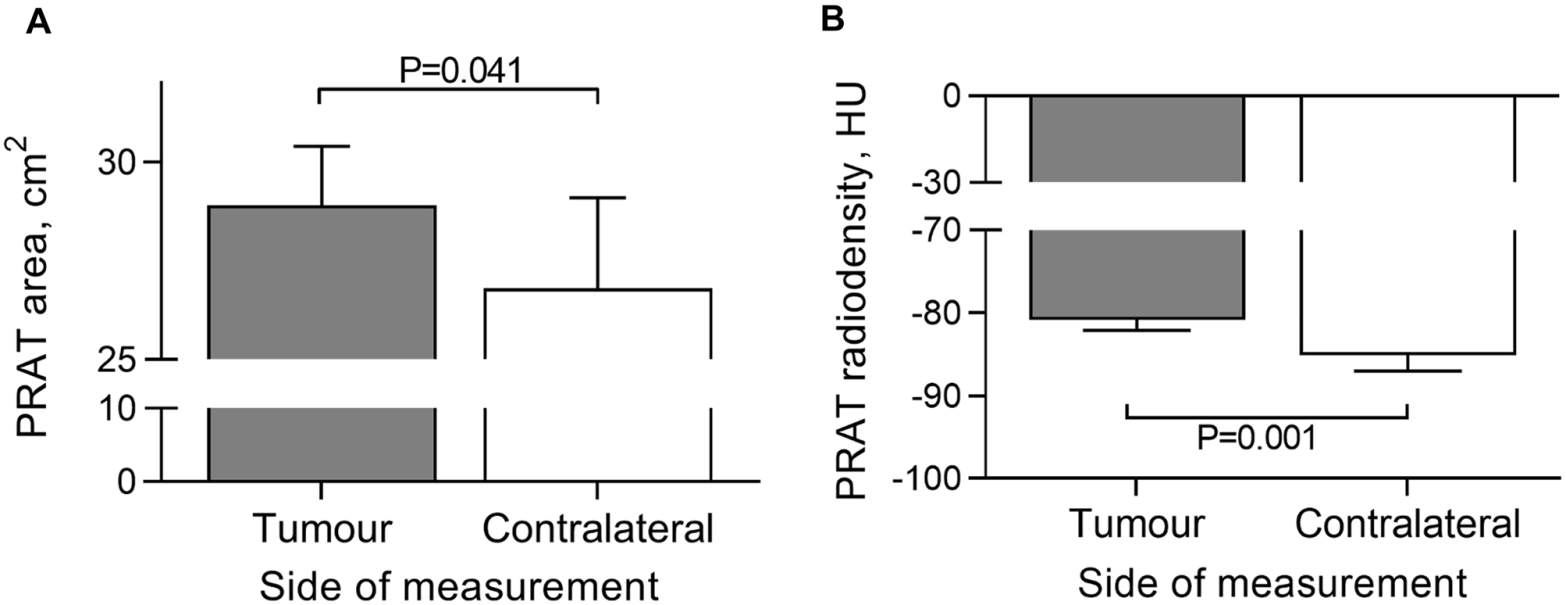
Within subject comparison of perirenal adipose tissue area and radiodensity. (**A**) perirenal adipose tissue area; (**B**) perirenal adipose tissue radiodensity. HU, Hounsfield Units. Mann Whitney test was used to compare groups. PRAT, perirenal adipose tissue.

**Figure 3:**
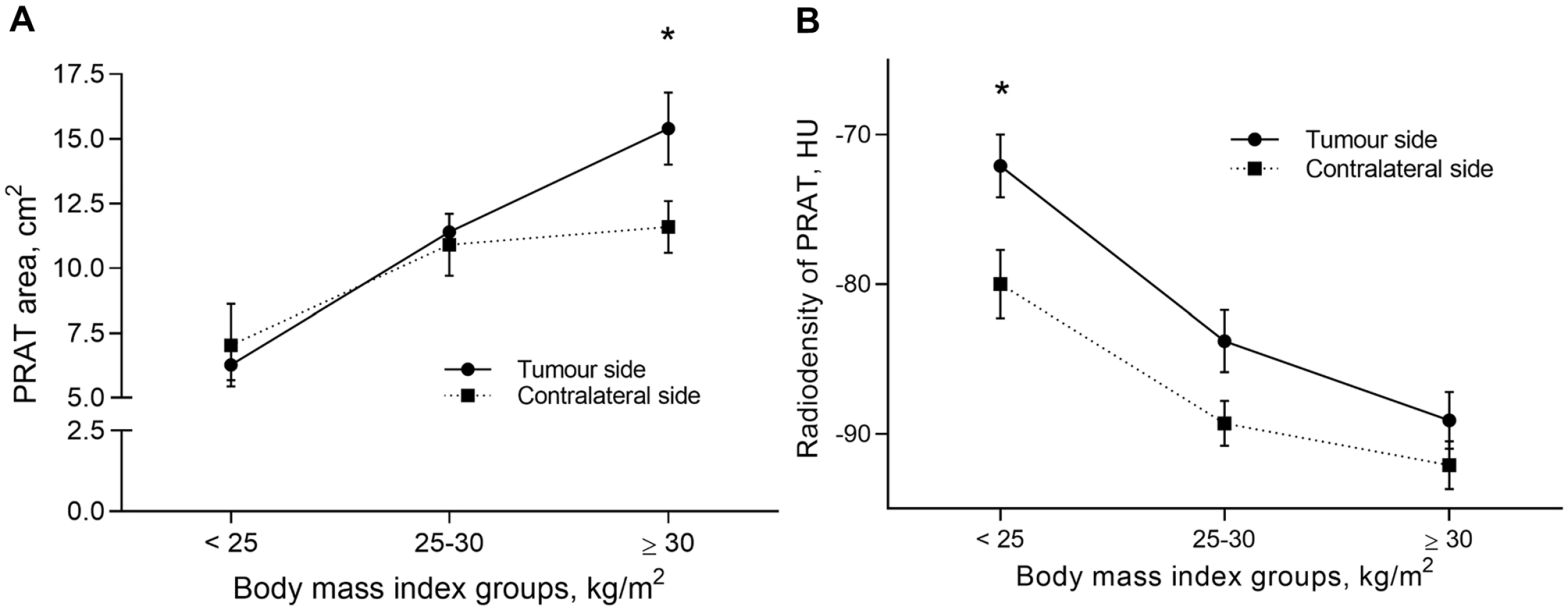
Tumour-side and contralateral perirenal adipose tissue areas and radiodensity by body mass index categories. BMI, body mass index; HU, Hounsfield Units. The Mann Whitney test was used to compare groups within each BMI category. (**A**) perirenal adipose tissue area (*P < 0.05); (**B**) perirenal adipose tissue radiodensity (*P < 0.05). PRAT, perirenal adipose tissue.

PRAT areas and radiodensities were measured in both kidneys and compared between tumour and contralateral side (Fig. 2), uncovering significantly higher areas on tumour side (*P* = 0.041). The PRAT radiodensity was higher in tumour side (*P* = 0.001). None of the participants had bilateral tumours, while tumour frequency was similar, with 43% right- and 57% left-sided. Areas and radiodensities were compared between tumour and contralateral PRAT by BMI categories (Fig. 3). PRAT area increases by BMI group, being higher in tumour versus contralateral, particularly in the obese group with BMI > 30 (*P* = 0.013). Conversely, radiodensity decreased as BMI increases, with significantly higher density in the tumour side of lean subjects (*P* = 0.006).

### Adipocytes size and gene expression of browning genes in adipose tissue samples

**Figure 4:**
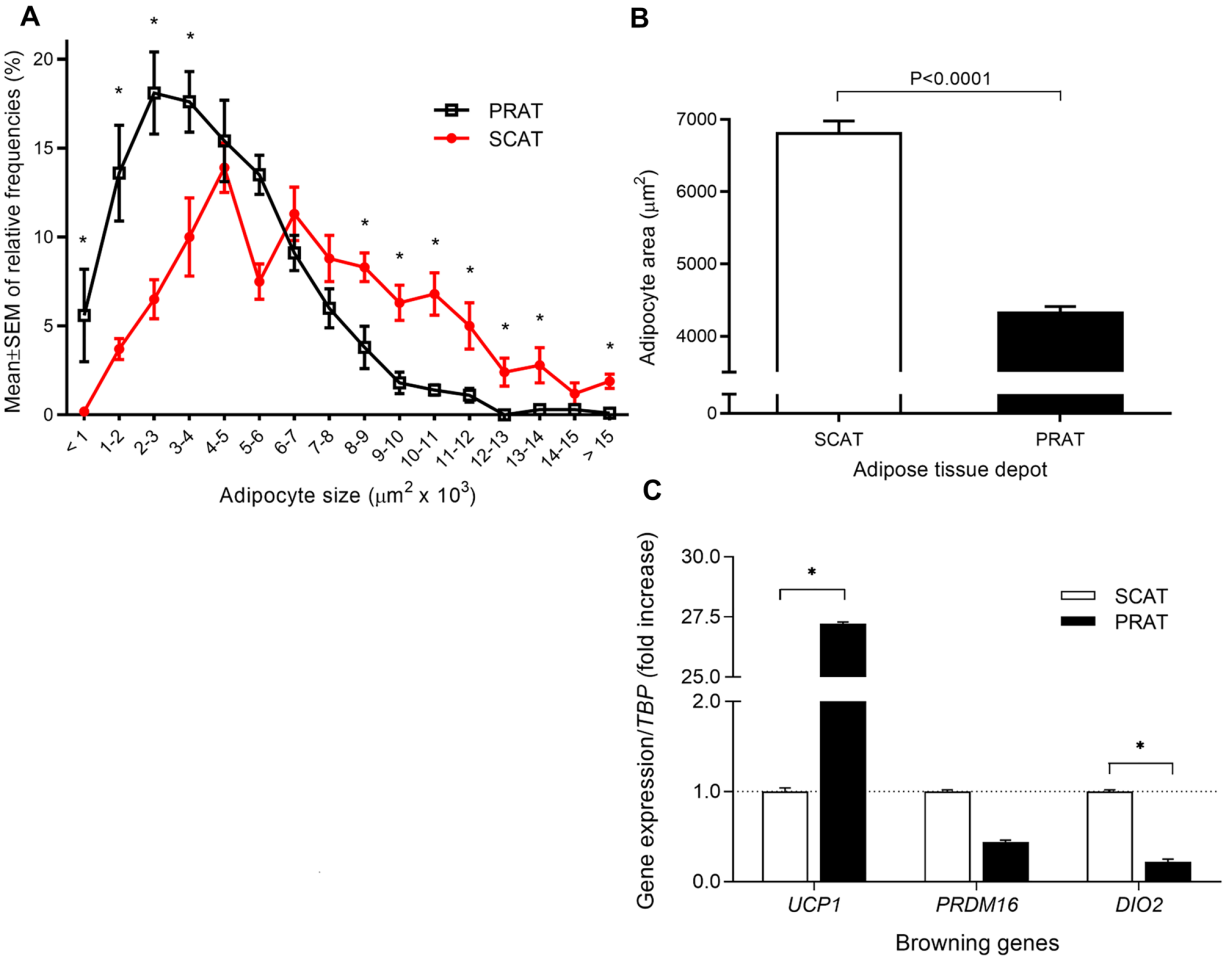
Adipocyte size (area) and expression of browning genes among perirenal and subcutaneous adipose tissue. Findings from histological evaluation of adipocyte’s areas from each depot (n = 8 PRAT and n = 5 SCAT), using midpower field (200x). At least 10 random microphotographs were taken from each specimen, resulting in a mean number of 160 adipocytes for perirenal and 94 for subcutaneous adipose tissue observed per patient. (**A**) Comparison of relative frequencies of adipocytes along adipocyte size area intervals, between SCAT and PRAT Mann Whitney test was used for comparison among depots within each adipocyte size interval. Data is presented as mean ± SEM from each subject distribution of adipocytes by size interval (**B**) Comparison of mean adipocyte area among subcutaneous and perirenal adipose tissue depots, using Mann–Whitney test. Mean ± SEM are used to depict data. (**C**) comparison of *UCP1, DIO2* and *PRDM16 gene expression* between tumour-sided perirenal fat matched with subcutaneous adipose tissue samples; data presented as fold-change and SEM. *P < 0.05. PRAT, perirenal adipose tissue; SCAT, subcutaneous adipose tissue.

**Figure 5:**
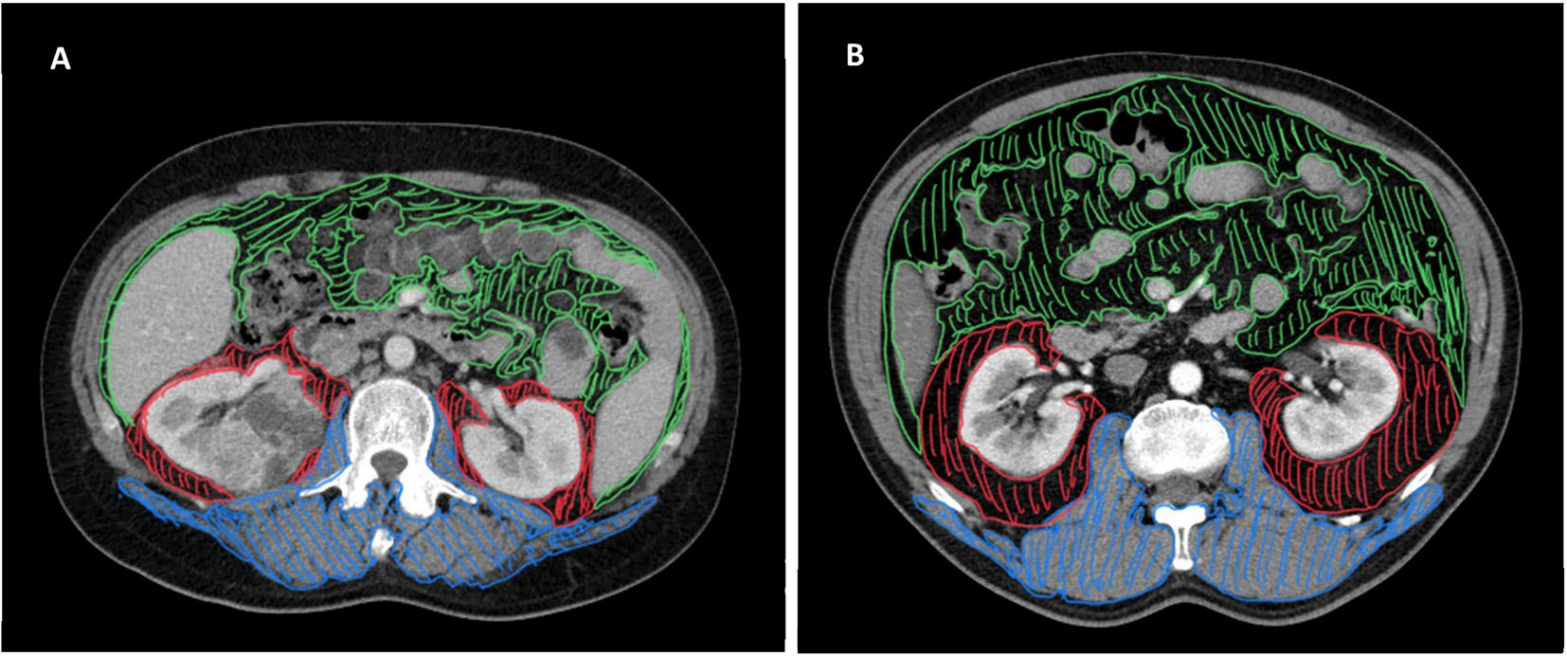
Representative examples of adiposity measures using CT scan. Axial images at the level of renal hilum from a non-obese (**A**) and an obese patient with RCCa (**B**). VAT is represented in green, PRAT in red and skeletal muscles in blue. VAT, visceral adipose tissue area; PRAT, perirenal adipose tissue; CT, computed tomography.

Adipocyte size was histologically compared among fat depots using the mean of adipocyte’s areas relative frequencies from participating individuals (Fig. 4A,B). Comparison of relative frequencies of adipocytes along adipocyte size area intervals, between SCAT and PRAT, revealed that low size adipocytes are overrepresented and large adipocytes underrepresented in PRAT compared with SCAT. In tumour-sided PRAT matched with SCAT samples, *UCP1* expression was 27-fold upregulated (*P* = 0.026), whereas *DIO2* and *PRDM16* were downregulated (*P* = 0.048 and *P* > 0.05) in PRAT (Figs. 4C, 5).

### Association of adiposity and SkM measures with RCCa aggressiveness (Fuhrman grade and pathological stage)

To evaluate obesity measures and SkM area in association with RCCa aggressiveness indicators (Fuhrman grade and pathological stage), we used multivariate logistic regression with adjustment for age, BMI, gender, tobacco, and hypertension (Table 2). Patients with BMI > 30 and in top tertiles of SkM area were associated with protection for advanced stage of disease, whereas the top tertiles of VAT and SkM areas associated with higher Fuhrman grade. No association was observed for PRAT areas, ratios or radiodensities.

**Table 2:** Association of tertiles of obesity measures and skeletal muscle area with risk for renal cell cancer aggressiveness features.

Variables	Stage (I-II vs. III-IV)		Furhman (1–2 vs. 3–4)	
	OR (95%CI)	P	OR (95%CI)	P
**BMI, kg.m**^**-2**^				
< 25	Referent		Referent	
25—30	0.9 (0.4–2.3)	0.878	1.8 (0.7–4.9)	0.252
> 30	0.2 (0.03–0.8)	0.028	1.2 (0.4–3.8)	0.761
**VAT area, cm**^**2**^				
T1	Referent		Referent	
T2	0.7 (0.2–2.6)	0.587	0.5 (0.1–2.1)	0.352
T3	0.9 (0.2–4.2)	0.942	0.2 (0.04–0.99)	0.049
**SkM area**				
T1	Referent		Referent	
T2	0.1 (0.04–0.6)	0.005	0.6 (0.2–1.9)	0.408
T3	0.3 (0.1–0.9)	0.038	0.2 (0.04–0.7)	0.015

BMI, body mass index; OR, odds ratio; 95%CI, 95% confidence interval; VAT, visceral adipose tissue; SkM, skeletal muscle area of the Erectorspinae, Psoas and Quadratus Lomborum muscles. Logistic regression with adjustment for age (< median vs. > median age), BMI groups (< 25 vs. 25–30 vs. > 30 kg.m^-2^), gender (male vs. female), tobacco (yes vs. no) and hypertension (yes vs. no).

### Empirical and multivariate models to determine the association of PRAT with progression-free survival and overall survival

Kaplan–Meier plots with Log-rank tests and multivariate Cox hazards estimates were used to determine the association of PRAT with both primary and secondary endpoints (progression-free survival and overall survival). A shorter time-to-PFS was observed for advanced stage and grade of disease, and for lower tertiles of tumour-sided PRAT area (Log-Rank test, all *P* < 0.05) (Supplementary table 3). Then, to test the robustness of independent variables with *P* < 0.10, a multivariate Cox regression model was estimated. The estimated risk for all-cause mortality was decreased for subjects in the top tertile of PRAT area of the tumour side (HR = 0.3, 95CI = 0.1–0.7, *P* = 0.009) (Table 3). Increased PRAT area from contralateral side was a significant independent predictor of PFS, with protective effect for disease progression (HR = 0.3, 95CI = 0.1–0.8, *P* = 0.019).

**Table 3:** Multivariate Cox regression models estimates for progression-free survival and overall survival.

	Progression-free survival		Overall survival	
	HR (95%CI)	P	HR (95%CI)	P
Median age, top group	11.1 (2.0–61.2)	0.006	22.7 (2.6–195.2)	0.004
Stage, III-IV	38.6 (6.5–228.0)	< 0.001	5.8 (1.8–18.3)	0.003
Furhman grade, 3–4	12.7 (3.2–50.1)	< 0.001	–	–
Surgical margins, negative	–	–	0.1 (0.02–0.4)	0.001
PRATt, top tertile	–	–	0.3 (0.1–0.7)	0.009
PRATcl, top tertile	0.3 (0.1–0.8)	0.019	–	–

PRATt, perirenal adipose tissue tumor side; PRATcl, perirenal adipose tissue contralateral side.

The Cox regression models for PFS and OS were fitted to the subgroup of Clear Cell RCCa (n = 91), confirming PRAT in tumour side as an independent prognosticator of OS (HR = 0.2, 95CI = 0.04–0.6, *P* = 0.010), and PRAT in contralateral side as an independent predictor of PFS (HR = 0.4, 95CI = 0.1–0.9, *P* = 0.028).

## Discussion

Obesity has been strongly associated with risk for developing RCCa, despite paradoxically related with better prognosis^22^. Current findings from our work support that an adiposity measure, the PRAT area evaluated by CT, associates with improved RCCa prognosis.

The most appropriate measure of obesity remains debatable, particularly when in association with cancer risk. BMI and waist measures are useful but inaccurate to estimate obesity. A more appropriate measure has been VAT area, a particular endocrine depot that secretes adipokines that might impact cancer, quantified through CT. Local fat tissue depots seem to crosstalk with VAT and exert a paracrine pro-tumoral effect^23–25^. Visceral obesity as assessed by VAT area has been strongly associated with better prognosis, both in localized and advanced renal cell carcinoma^26,27^. We found that VAT area and BMI were good predictors of PRAT area in the tumour side, explaining respectively 58 and 48% of variation. Notably, the top tertiles of PRAT areas in tumour side and contralateral side protects from death and disease progression, overall and when fitted to clear cell RCCa histological subtype. The positive correlation between PRAT and other RCCa-associated measures such as BMI and VAT area further support PRAT area measurement as a proxy of obesity and a tool to evaluate predictive and prognostic risks in RCCa patients. Future studies including other obesity measures, such as waist and waist-to-hip ratio measures, warrant additional information on their association with PRAT.

Besides soluble- and cell-mediated systemic effects, the obesity-cancer link is likely to be shaped by local fat depots. The PRAT depot, adjacent to the kidney, among Gerota and renal fascia, is metabolically active and morphological and functionally distinctive. The immediacy amongst these two organs facilitates interaction, providing another plausible explanation for obesity paradox in RCCa. Concordantly, histopathological assessment of the invasion of PRAT by RCCa cells in surgical specimens evidenced that prognostic significance depends on tumor size, particularly if at pT3a stage^28^. Moreover, patients bearing pT3a tumours with PRAT invasion seem to have better prognosis than those with sinus fat invasion^29,30^. Although few reports suggest that confounding factors accounted for the positive effect of obesity in prognosis^31^, a recent large molecular study revealed the transcriptional landscape of PRAT and tumour in obesity-associated RCCa^19^, further eliciting a biological plausibility for PRAT in RCCa. In vitro experiments using conditioned medium from PRAT primary cultures (collected near versus away from the tumour, and of RCCa patients versus non-malignant subjects), revealed distinct secretory profiles and function in RCCa cell lines^18,20^. Accordingly, heterogeneity of PRAT has been proposed in human adults^17^ eventually due to the presence of dormant BAT^32^. It is known that PRAT from patients with RCCa express significantly more *UCP1* and is characterized by smaller adipocytes in comparison with PRAT from subjects without malignancy^33,34^. We analyzed histological sections of PRAT and SCAT from RCCa patients, which evidenced an elevated *UCP1* expression and small adipocyte area, supporting the presence of a *brown-like* fat tissue in PRAT compared to SCAT. Despite recent in vitro findings that browning PRAT-secreted factors might stimulate epithelial-to-mesenchymal transition in normal and malignant renal cells and likely contribute to tumour development^34^, a recent report revealed for the first time the involvement of BAT activation in tumour suppression, providing a novel therapeutic concept for cancer treatment involving switching on BAT activity^35^.

Immunoinflammation has been described as a hallmark of cancer, which has been associated with RCCa development and progression^36^. White adipose tissue was shown to secrete adipokines required for regulation of glucose and lipid homeostasis, as well as inflammation. In white adipose tissue of overweight and obese, inflammatory foci consist of dead adipocytes encircled by macrophages that are activated through fatty acids released by obesity-associated lipolysis, stimulating NF-κB pathway and proinflammatory mediators. However, limited evidence elucidates PRAT involvement in the regulation of local and systemic immunoinflammatory mechanisms^37^. A key feature specific in PRAT is the brown-like phenotype and cancer-induced WAT browning, which upon activation are known to reduce inflammation^35^, thereby reducing cancer aggressiveness. Future studies are warranted to characterize inflammatory profile of PRAT and uncover the crosstalk between tumour and PRAT microenvironments.

Imaging techniques, such as ultrasonography, computed tomography or magnetic resonance imaging, have been applied to study PRAT depot in association with several types of malignancies and nephropaties. Most studies used PRAT thickness, measured as the distance between kidney and Gerota fascia, which was described as an estimate of PRAT mass^8^. However, controversial findings from association studies revealed that higher PRAT thickness associated with decreased time to progression-free survival (PFS) in RCCa^10^, but with lower rates of PFS in ovarian cancer^38^. Moreover, PRAT thickness has been linked to postoperative complications in gastric cancer^39^, while lack of association was observed with colorectal cancer survival^40^. We provide evidence that advanced stage and lower PRAT areas of contralateral and tumour sided are related with shorter time-to-PFS and time-to-death, still after multivariate analysis. These findings enlightened the hypothesis of protection provided by larger PRAT depots against more aggressive disease and complies with the obesity paradox^4,41^. Further studies in larger population are warranted to strengthen current results.

Besides the quantification of PRAT we evaluated the quality of PRAT deposit in CT imaging through radiodensity measurement. Beyond higher PRAT areas, we observed increased densities in PRAT on the tumour side compared to the contralateral non-tumour kidney. Notably, while in the PRAT area the difference between tumour and contralateral side was higher within the obese group of patients, the PRAT radiodensity was significantly higher on tumour side versus non-tumour for normal weight individuals. In a recent work, Din et al.^42^ reported that radiodensity was well correlated to BAT exposure to cold temperatures, upon activation of cell activity and thermogenesis. Radiodensity of adipose tissue, measured as attenuation across all voxels within the structure selected in a cross-sectional CT image, might have a biological significance representing metabolic activity in the fat tissue^43^, and reflect the lipid content and the size of adipocytes^7^. Taken together, these findings suggest that higher density and area in the tumour side of PRAT likely reflect a crosstalk among adipose tissue and tumour. Future studies are warranted in RCCa specimens at the interface of the invasive front of tumour with PRAT, particularly in stage pT3a-b.

Skeletal muscle index and radiodensity have been associated with clinical outcomes of some cancer types, including RCCa, where lower SkM index and radiodensity were linked to mortality in metastatic RCCa^44^. In agreement, increased SkM area in our population of RCCa subjects eligible for nephrectomy was associated with protection from advanced disease in univariate analysis.

Here, when obesity is classified according to BMI, larger PRAT areas and lower fat radiodensity are observed in overweight/obese patients, particularly on lower tumour stages when compared to normal weight. Taken together, this finding agrees with the obesity paradox^4,45–47^. Larger PRAT area might be associated with less metabolic activity (as shown by decreased density), and subsequently conferring less pro-tumoral paracrine signals to malignant cells.

Perirenal fatness seems to correlate well with hypertension^48,49^. The adipose afferent reflex neural circuit, together with locally produced adipokines^25,50^, confer PRAT with unique features to promote hypertension, which is in correlation with increased thickness^48,51^. Our data further support this relationship, since hypertensive patients have higher areas of PRAT, both in tumour and contralateral kidneys. Given the tight association between hypertension and obesity, and between hypertension and renal cancer, we hypothesize that increased PRAT mass may mediate this link through depot-specific adipokine production and neural circuits.

In this study, clinicopathological characteristics of participants were predominantly low grade, thus explaining the rather low number of fatal or recurrent events, which limit our ability to infer the influence of obesity and fat depots on RCCa prognosis. Furthermore, only a small number of patients with less common subtypes of renal cancer are included, hence limiting generalizability. A larger cohort study is warranted to further validate current findings.

We propose PRAT as a possible tool, well correlated with other measures of obesity such as VAT area and BMI, that will improve determination of obesity and contribute to assess risks of disease progression and mortality in renal cancer patients. Presented data supports the obesity paradox in RCCa, assumed that larger PRAT areas seem to protect from disease progression and death. Larger areas and decreased density of tumor-sided PRAT likely represent the ultimate molecular activity from adipose cell-tumour cell crosstalk. Since we found hypertensive patients had larger and more dense PRAT depots on the tumour side, a common mechanistic linkage seems plausible.

## Supplementary Information

Supplementary Information 1.

Supplementary Information 2.

Supplementary Information 3.

Supplementary Information 4.

## Data Availability

The datasets used and/or analyzed during the current study available from the corresponding author on reasonable request.
