# Supplementary material for: A hint for the obesity paradox and the link between obesity, perirenal adipose tissue and Renal Cell Carcinoma progression
Source: Sci Rep. 2022 Nov 19;12:19956. doi: 10.1038/s41598-022-24418-9 (PMC9675816; doi:10.1038/s41598-022-24418-9)
Supplement: Supplementary file 1 — Supplementary Information 1. [file 41598_2022_24418_MOESM1_ESM.docx]

Supplementary table 1. Descriptive data of computed tomography scan measures at diagnosis

| **Variables** | **N** | **Median** | **IQR** | **Mean** | **SEM** |
| --- | --- | --- | --- | --- | --- |
| *Areas, cm^2^* | *137* |  |  |  |  |
| Visceral adipose tissue |  | 172.5 | 74.3 - 227.4 | 164.8 | 8.2 |
| Perirenal adipose tissue, tumor side |  | 27.2 | 15.3 - 39.6 | 28.9 | 1.5 |
| Perirenal adipose tissue,contralateral side |  | 21.3 | 11.1 - 35.1 | 26.8 | 2.3 |
| Skeletal muscle * |  | 62.6 | 50.6 - 75.3 | 71.3 | 6.3 |
| *Ratios of areas* | *137* |  |  |  |  |
| PRAT/Kidney, tumor side |  | 1.2 | 0.7 - 2.2 | 1.6 | 0.1 |
| PRAT/Kidney, contralateral side |  | 1.2 | 0.6 - 1.8 | 1.6 | 0.2 |
| PRAT, tumor side/contralateral side |  | 1.2 | 0.9 - 1.6 | 1.6 | 0.1 |
| *Radiodensity, Hounsfield units* | *131* |  |  |  |  |
| PRAT, tumor side |  | -85.0 | (-92.0) – (-70.5) | -80.8 | 1.3 |
| PRAT, contralateral side |  | -89.0 | (-97.0) – (-79.0) | -86.7 | 1.2 |
| Skeletal muscle * |  | 35.0 | 26.0 - 42.0 | 33.9 | 1.1 |
| PRAT, tumor minus contralateral side |  | -4.0 | -13.0 - 0.0 | -6.3 | 1.1 |

CT, computed tomography scan; PRAT, perirenal adipose tissue; * skeletal muscle area and radiodensity of the Erectorspinae, Psoas and Quadratus Lomborum muscles. IQR, interquartile range; SEM, standard error of mean.
