# Supplementary material for: A hint for the obesity paradox and the link between obesity, perirenal adipose tissue and Renal Cell Carcinoma progression
Source: Sci Rep. 2022 Nov 19;12:19956. doi: 10.1038/s41598-022-24418-9 (PMC9675816; doi:10.1038/s41598-022-24418-9)
Supplement: Supplementary file 2 — Supplementary Information 2. [file 41598_2022_24418_MOESM2_ESM.docx]

Supplementary table 2. Association of demographic and clinical variables with adipose tissue, kidney and skeletal muscle measures on computed tomography scan

|  | Age at diagnosis, n (frequency) | | | Gender, n (frequency) | | | Tobacco, n (frequency) | | | Hypertension, n (frequency) | | |
| --- | --- | --- | --- | --- | --- | --- | --- | --- | --- | --- | --- | --- |
| **Tertiles of CT measures** | < 64.7 yrs | ≥ 64.7 yrs | P * | ♂ | ♀ | P * | No | Yes | P * | No | Yes | P * |
| ***Areas, cm^2^*** | | | | | | | | | | | | |
| Visceral adipose tissue  < 112.3  112.3 – 205.6  ≥ 205.6 | 26 (0.38)  24 (0.35)  18 (0.27) | 19 (0.27)  22 (0.32)  28 (0.41) | 0.188 | 20 (0.23)  30 (0.34)  37 (0.43) | 25 (0.50)  16 (0.32)  9 (0.18) | 0.002 | 30 (0.36)  27 (0.32)  27 (0.32) | 15 (0.28)  19 (0.36)  19 (0.36) | 0.667 | 27 (0.53)  12 (0.24)  12 (0.23) | 18 (0.21)  34 (0.40)  34 (0.39) | 0.001 |
| PRAT, tumor side  < 19.0  19.0 – 33.9  ≥ 33.9 | 29 (0.43)  23 (0.34)  16 (0.23) | 16 (0.23)  24 (0.35)  29 (0.42) | 0.023 | 20 (0.23)  33 (0.38)  34 (0.39) | 25 (0.50)  14 (0.28)  11 (0.22) | 0.005 | 30 (0.36)  27 (0.32)  27 (0.32) | 15 (0.28)  20 (0.38)  18 (0.34) | 0.646 | 30 (0.59)  13 (0.25)  8 (0.16) | 15 (0.17)  34 (0.40)  37 (0.43) | < 0.001 |
| PRAT, contralateral side  < 14.5  14.5 – 30.8  > 30.8 | 25 (0.37)  26 (0.38)  17 (0.25) | 19 (0.28)  21 (0.30)  29 (0.42) | 0.107 | 18 (0.21)  34 (0.39)  35 (0.40) | 26 (0.52)  13 (0.26)  11 (0.22) | 0.001 | 14 (0.26)  20 (0.38)  19 (0.36) | 30 (0.36)  27 (0.32)  27 (0.32) | 0.521 | 19 (0.22)  34 (0.40) | 25 (0.49)  13 (0.26)  13 (0.25) | 0.005 |
| Skeletal muscle  < 55.7  55.7 – 71.2  ≥ 71.2 | 18 (0.26)  25 (0.37)  25 (0.37) | 27 (0.39)  21 (0.30)  21 (0.31) | 0.288 | 13 (0.15)  31 (0.36)  43 (0.49) | 32 (0.64)  15 (0.30)  3 (0.06) | < 0.001 | 13 (0.25)  16 (0.30)  24 (0.45) | 32 (0.38)  30 (0.36)  22 (0.26) | 0.059 | 32 (0.37)  26 (0.30)  28 (0.33) | 13 (0.26)  20 (0.39)  18 (0.35) | 0.336 |
| ***Ratios of areas*** | | | | | | | | | | | | |
| PRAT/Kidney, tumor side  < 0.9  0.9 – 1.8  ≥ 1.8 | 23 (0.34)  24 (0.35)  21 (0.31) | 22 (0.32)  22 (0.32)  25 (0.36) | 0.799 | 29 (0.33)  32 (0.37)  26 (0.30) | 16 (0.32)  14 (0.28)  20 (0.40) | 0.424 | 30 (0.36)  25 (0.30)  29 (0.34) | 15 (0.28)  21 (0.40)  17 (0.32) | 0.462 | 29 (0.57)  14 (0.27)  8 (0.16) | 16 (0.19)  32 (0.37)  38 (0.44) | < 0.001 |
| PRAT/Kidney, contralateral side  < 0.7  0.7 – 1.6  ≥ 1.6 | 28 (0.34)  26 (0.58)  14 (0.21) | 18 (0.26)  19 (0.28)  32 (0.46) | 0.006 | 22 (0.25)  34 (0.39)  31 (0.36) | 24 (0.48)  11 (0.22)  15 (0.30) | 0.018 | 28 (0.33)  27 (0.32)  29 (0.35) | 18 (0.34)  18 (0.34)  17 (0.32) | 0.954 | 25 (0.49)  16 (0.31)  10 (0.20) | 21 (0.24)  29 (0.34)  36 (0.42) | 0.005 |
| PRAT, tumor side/contralateral side  < 1.0  1.0 – 1.5  ≥ 1.5 | 19 (0.28)  27 (0.40)  22 (0.32) | 26 (0.38)  20 (0.29)  23 (0.33) | 0.342 | 27 (0.31)  33 (0.38)  27 (0.31) | 18 (0.36)  14 (0.28)  18 (0.36) | 0.499 | 28 (0.33)  28 (0.33)  28 (0.34) | 17 (0.32)  19 (0.36)  17 (0.32) | 0.955 | 17 (0.33)  20 (0.39)  14 (0.28) | 28 (0.33)  27 (0.31)  31 (0.36) | 0.523 |
| ***Radiodensity, Hounsfield Units*** | | | | | | | | | | | | |
| PRAT, tumor  < -90.3  -90.3 - -76  ≥ -76 | 23 (0.35)  23 (0.36)  19 (0.29) | 21 (0.31)  24 (0.35)  23 (0.34) | 0.808 | 29 (0.34)  30 (0.35)  27 (0.31) | 15 (0.32)  17 (0.36)  15 (0.32) | 0.977 | 26 (0.33)  33 (0.41)  21 (0.26) | 18 (0.34)  14 (0.26)  21 (0.40) | 0.149 | 10 (0.20)  19 (0.39)  20 (0.41) | 34 (0.41)  28 (0.33)  22 (0.26) | 0.047 |
| PRAT, contralateral  < -94.0  -94.0 - -83.0  ≥ -83.0 | 22 (0.35)  18 (0.29)  23 (0.36) | 26 (0.38)  24 (0.35)  18 (0.27) | 0.447 | 27 (0.31)  28 (0.33)  31 (0.36) | 21 (0.47)  14 (0.31)  10 (0.22) | 0.157 | 36 (0.46)  26 (0.33)  16 (0.21) | 12 (0.23)  16 (0.30)  25 (0.47) | 0.002 | 13 (0.28)  17 (0.36)  17 (0.36) | 35 (0.42)  25 (0.30)  24 (0.28) | 0.279 |
| PRAT, tumor minus contralateral  < 0.0  0.0 – 8.0  ≥ 8.0 | 24 (0.38)  21 (0.33)  18 (0.29) | 20 (0.30)  26 (0.38)  22 (0.32) | 0.575 | 32 (0.37)  33 (0.38)  21 (0.24) | 12 (0.27)  14 (0.31)  19 (0.42) | 0.107 | 24 (0.31)  27 (0.35)  27 (0.34) | 20 (0.38)  20 (0.38)  13 (0.24) | 0.451 | 17 (0.36)  14 (0.30)  16 (0.34) | 27 (0.32)  33 (0.39)  24 (0.29) | 0.549 |
| Skeletal muscle  < 29.0  29.0 – 40.0  ≥ 40.0 | 12 (0.19)  19 (0.30)  32 (0.51) | 33 (0.48)  26 (0.38)  10 (0.14) | < 0.001 | 23 (0.27)  30 (0.35)  32 (0.38) | 22 (0.47)  15 (0.32)  10 (0.21) | 0.047 | 31 (0.39)  27 (0.34)  22 (0.27) | 14 (0.27)  18 (0.35)  20 (0.38) | 0.288 | 10 (0.21)  16 (0.33)  22 (0.46) | 35 (0.42)  29 (0.34)  20 (0.24) | 0.014 |

Data presented as n (relative frequency). * P-values calculated using Pearson chi-square. ♂, male, ♀, female; CT, computed tomography scan; PRAT, perirenal adipose tissue; SkM, skeletal muscle area of the Erectorspinae, Psoas and Quadratus Lomborum muscles; Ratio of ratios=Ratio of PRAT/Kidney,tumor side / Ratio of PRAT/Kidney, contralateral side
