# Supplementary material for: A hint for the obesity paradox and the link between obesity, perirenal adipose tissue and Renal Cell Carcinoma progression
Source: Sci Rep. 2022 Nov 19;12:19956. doi: 10.1038/s41598-022-24418-9 (PMC9675816; doi:10.1038/s41598-022-24418-9)
Supplement: Supplementary file 3 — Supplementary Information 3. [file 41598_2022_24418_MOESM3_ESM.docx]

Supplementary table 3. Overall survival and progression free survival Kaplan Meier estimates with Log-Rank tests of clinicopathological variables and computed tomography measures

|  |  | Progression-free survival | |  | Overall survival | |
| --- | --- | --- | --- | --- | --- | --- |
|  | N | M (95CI) | P |  | M (95CI) | P |
| Median age  < 64.7  ≥ 64.7 | 68  69 | 91.0 (86.0-95.9)  89.0 (79.8-98.2) | 0.086 |  | 98.6 (95.1-102.0)  84.3 (74.8-93.8) | **0.002** |
| Gender  Male  Female | 87  50 | 93.0 (86.1-100.0)  89.0 (81.9-96.1) | 0.659 |  | 92.5 (85.5-99.5)  87.6 (80.3-95.0) | 0.952 |
| Obesity, WHO  NW  OW  OB | 50  52  35 | 83.6 (76.8-90.4)  91.5 (81.8-101.1)  90.4 (84.2-96.6) | 0.641 |  | 82.7 (74.7-90.6)  91.7 (81.8-101.5)  89.9 (83.0-96.8) | 0.561 |
| Tobacco  No  Yes | 84  53 | 87.2 (81.5-92.9)  93.5 (84.5-102.4) | 0.876 |  | 88.7 (83.7-93.8)  88.9 (79.1-98.7) | 0.252 |
| Hypertension  No  Yes | 51  86 | 78.9 (72.2-85.5)  94.1 (87.4-100.8) | 0.914 |  | 90.3 (82.9-97.7)  82.2 (77.0-87.3) | 0.213 |
| Stage of disease  I-II  III.IV | 109  28 | 101.7 (96.9-106.6)  61.6 (41.8-81.5) | **<0.001** |  | 96.3 (90.2-102.4)  76.3 (59.5-93.1) | **0.001** |
| Furhman  1-2  3-4 | 109  28 | 100.4 (96.5-104.4)  62.0 (45.7-78.3) | **<0.001** |  | 96.5 (90.9-102.2)  70.6 (56.3-84.8) | **0.006** |
| Surgical margins  Positive  Negative | 7  130 | 72.4 (51.8-93.1)  94.2 (88.4-100.1) | 0.268 |  | 59.3 (25.3-93.4)  95.0 (89.4-100.7) | **< 0.001** |
| *Areas, cm^2^* |  |  |  |  |  |  |
| Visceral adipose tissue tertiles  < 112.3  112.3 – 205.6  ≥ 205.6 | 45  46  46 | 74.8 (66.0-83.7)  92.8 (83.5-102.1)  91.4 (86.1-96.8) | 0.257 |  | 76.0 (68.2-83.7)  90.2 (80.1-100.4)  93.6 (86.3-100.9) | 0.439 |
| Perirenal adipose tissue tertiles, tumor  < 19.0  19.0 – 33.9  ≥ 33.9 | 45  47  45 | 81.3 (75.4-87.1)  77.5 (67.0-88.0)  100.2 (94.5-105.9) | **0.013** |  | 81.2 (75.3-87.0)  73.9 (63.3-84.6)  100.8 (95.1-106.5) | **0.002** |
| Perirenal adipose tissue tertiles,contralateral  < 14.5  14.5 – 30.8  > 30.8 | 44  47  46 | 78.7 (71.0-86.4)  77.1 (68.6-85.5)  100.3 (94.8-105.8) | 0.059 |  | 79.9 (73.7-86.1)  75.4 (66.2-84.6)  96.7 (88.9-104.6) | 0.173 |
| Skeletal muscle tertiles  < 55.7  55.7 – 71.2  ≥ 71.2 | 45  46  46 | 92.1 (81.8-102.3)  86.9 (79.3-94.5)  98.5 (91.4-105.7) | 0.436 |  | 87.8 (77.4-98.2)  84.1 (75.7-92.5)  100.5 (94.3-106.7) | 0.128 |
| *Ratios of areas* |  |  |  |  |  |  |
| PRAT/Kidney tertiles, tumor side  < 0.9  0.9 – 1.8  ≥ 1.8 | 45  46  46 | 95.1 (84.3-105.8)  89.6 (79.1-100.1)  96.0 (89.1-102.9) | **0.026 *** |  | 90.4 (79.7-101.0)  90.2 (78.5-101.8)  93.7 (85.7-101.7) | 0.577 |
| PRAT/Kidney tertiles, contralateral side  < 0.7  0.7 – 1.6  ≥ 1.6 | 46  45  46 | 75.4 (66.9-83.9)  96.1 (87.7-104.5)  97.0 (89.0-104.9) | 0.477 |  | 77.5 (69.9-85.0)  93.9 (83.5-104.2)  91.7 (83.2-100.2) | 0.954 |
| PRAT tertiles, tumor side/contralateral side  < 1.0  1.0 – 1.5  ≥ 1.5 | 45  47  45 | 99.3 (92.2-106.4)  92.0 (81.6-102.3)  75.4 (66.7-84.1) | 0.350 |  | 94.4 (86.0-102.8)  92.1 (81.9-102.2)  76.2 (68.4-83.9) | 0.534 |
| *Radiodensity, Hounsfield units* |  |  |  |  |  |  |
| PRAT tertiles, tumor side  < -90.3  -90.3 - -76  ≥ -76 | 44  47  42 | 102.2 (96.6-107.8)  89.7 (80.2-99.1)  88.1 (75.7-106.5) | 0.056 |  | 94.8 (85.5-104.0)  94.3 (85.8-102.9)  87.1 (76.3-98.0) | 0.248 |
| PRAT tertiles, contralateral side  < -94.0  -94.0 - -83.0  ≥ -83.0 | 48  42  41 | 98.1 (90.5-105.7)  90.5 (80.8-100.3)  88.9 (75.7-102.1) | 0.250 |  | 98.3 (90.9-105.7)  84.8 (74.2-95.5)  92.3 (82.3-102.3) | 0.311 |
| PRAT tertiles, tumor minus contralateral side  < 0.0  0.0 – 8.0  ≥ 8.0 | 45  43  43 | 85.8 (72.8-98.8)  94.6 (87.0-102.2)  98.4 (89.8-107.0) | 0.097 |  | 86.1 (75.3-97.0)  96.0 (89.0-103.1)  94.6 (85.0-104.2) | **0.032*** |
| Skeletal muscle tertiles  < 29.0  29.0 – 40.0  ≥ 40.0 | 45  45  42 | 95.2 (86.0-104.4)  87.3 (82.8-91.7)  88.4 (76.3-100.5) | 0.198 |  | 93.2 (83.8-102.7)  80.0 (72.1-87.8)  94.2 (84.3-104.1) | 0.670 |

CT, computed tomography scan; PRAT, perirenal adipose tissue; SkM, skeletal muscle area of the Erectorspinae, Psoas and Quadratus Lomborum muscles; Ratio of ratios, Ratio of PRAT/Kidney, tumor side / Ratio of PRAT/Kidney, contralateral side. * Breslow test for equality of survival distributions.
