# Supplementary material for: A hint for the obesity paradox and the link between obesity, perirenal adipose tissue and Renal Cell Carcinoma progression
Source: Sci Rep. 2022 Nov 19;12:19956. doi: 10.1038/s41598-022-24418-9 (PMC9675816; doi:10.1038/s41598-022-24418-9)
Supplement: Supplementary file 4 — Supplementary Information 4. [file 41598_2022_24418_MOESM4_ESM.docx]

Supplementary table 4. Primers used for qPCR

| Gene | Forward primer (5’-3’) | Reverse primer (5’-3’) |
| --- | --- | --- |
| *UCP1* | TGCCCAACTGTGCAATGAA | TCGCAAGAAGGAAGGTACCAA |
| *DIO2* | CCTCCTCGATGCCTACAAAC | GCTGGCAAAGTCAAGAAGGT |
| *PRDM16* | GAGGAGGACGATGAGGACAG | GAGGAGGACGATGAGGACAG |
| *TBP* | TCAAACCCAGAATTGTTCTCCTTAT | CCTGAATCCCTTTAGAATAGGGTAGA |
